# CRISPR-Cas in *Pseudomonas aeruginosa* provides transient population-level immunity against high phage exposures

**DOI:** 10.1093/ismejo/wrad039

**Published:** 2024-01-10

**Authors:** Bridget N J Watson, Loris Capria, Ellinor O Alseth, Benoit J Pons, Ambarish Biswas, Luca Lenzi, Angus Buckling, Stineke van Houte, Edze R Westra, Sean Meaden

**Affiliations:** Biosciences, University of Exeter, Penryn, Cornwall, TR10 9FE, United Kingdom; Biosciences, University of Exeter, Penryn, Cornwall, TR10 9FE, United Kingdom; Biosciences, University of Exeter, Penryn, Cornwall, TR10 9FE, United Kingdom; Center for Microbial Dynamics and Infection, Georgia Institute of Technology, Atlanta, GA 30332, United States; Biosciences, University of Exeter, Penryn, Cornwall, TR10 9FE, United Kingdom; Department of Microbiology and Immunology, University of Otago, Dunedin, 9059, Otago, New Zealand; Institute of Integrative Biology, University of Liverpool, Liverpool, Merseyside, L69 7BE, United Kingdom; Biosciences, University of Exeter, Penryn, Cornwall, TR10 9FE, United Kingdom; Biosciences, University of Exeter, Penryn, Cornwall, TR10 9FE, United Kingdom; Biosciences, University of Exeter, Penryn, Cornwall, TR10 9FE, United Kingdom; Biosciences, University of Exeter, Penryn, Cornwall, TR10 9FE, United Kingdom; Department of Biology, University of York, Wentworth Way, York, North Yorkshire YO10 3DB, United Kingdom

**Keywords:** CRISPR, evolution, phage, immunity, ecological dynamics

## Abstract

The prokaryotic adaptive immune system, CRISPR-Cas (clustered regularly interspaced short palindromic repeats; CRISPR-associated), requires the acquisition of spacer sequences that target invading mobile genetic elements such as phages. Previous work has identified ecological variables that drive the evolution of CRISPR-based immunity of the model organism *Pseudomonas aeruginosa* PA14 against its phage DMS3vir, resulting in rapid phage extinction. However, it is unclear if and how stable such acquired immunity is within bacterial populations, and how this depends on the environment. Here, we examine the dynamics of CRISPR spacer acquisition and loss over a 30-day evolution experiment and identify conditions that tip the balance between long-term maintenance of immunity versus invasion of alternative resistance strategies that support phage persistence. Specifically, we find that both the initial phage dose and reinfection frequencies determine whether or not acquired CRISPR immunity is maintained in the long term, and whether or not phage can coexist with the bacteria. At the population genetics level, emergence and loss of CRISPR immunity are associated with high levels of spacer diversity that subsequently decline due to invasion of bacteria carrying pilus-associated mutations. Together, these results provide high resolution of the dynamics of CRISPR immunity acquisition and loss and demonstrate that the cumulative phage burden determines the effectiveness of CRISPR over ecologically relevant timeframes.

## Introduction

Bacteria and Archaea encode a wide range of different defense strategies that protect against infection by their viruses. Of these, CRISPR-Cas (clustered regularly interspaced short palindromic repeats; CRISPR-associated) is the only system known to provide adaptive and heritable defense [1]. CRISPR-Cas systems rely on inserting sequences from viruses (and other mobile genetic elements), known as spacers, into CRISPR loci on the host genome, which function as a genetic memory to detect and destroy the virus upon reinfection (reviewed in [2]). The carriage of additional spacers is typically cost-free for the host [3], except if they target genes inserted into the host genome [4-8]. Bacteria and Archaea can thus accumulate phage-targeting spacers to increase their resistance range at very little or no cost [9]. Yet, spacers can also be lost [5], which may explain why most CRISPR arrays found in bacterial genomes are of moderate size, typically with 50 or fewer spacers [10]. Understanding the dynamics of spacer gain and loss following infection is therefore important for interpreting the patterns of arrays observed in comparative genomic studies (reviewed in Garrett [11]) and metagenomic analyses [12], and for understanding the consequences of CRISPR immunity for bacteria–phage coexistence. For example, high frequencies of spacer acquisition can drive rapid extinction of phage [13], as mutant “escape” phages are targeted by multiple spacers, whereas high frequencies of spacer loss may facilitate bacteria–phage coexistence [14].

A recent study using the *Pseudomonas aeruginosa* PA14 and phage DMS3vir model demonstrated that the initial infection dosage strongly predicts the frequency at which CRISPR immunity initially emerges, with high doses leading to higher frequencies of acquired CRISPR immunity and lower doses leading to relatively more surface-based resistance (i.e. loss of the phage receptor; referred to as SM herein). This is because acquisition of CRISPR immunity is infection dependent, and higher infection frequencies therefore lead to more cells acquiring CRISPR immunity [15]. However, high infection frequencies also amplify the infection-induced fitness cost of CRISPR immunity, which likely results from immune lag, where the initial redirection of host resources to viral reproduction prior to immune response is costly [16-18]. This in turn leads to the invasion of bacteria with SM resistance, which carries a fixed cost of resistance [16, 19]. The opposing effects of phage dose on the acquisition and selection of CRISPR immunity complicate our ability to predict the long-term population dynamics of CRISPR-immune bacteria. Since CRISPR-immune bacteria are much more effective in driving phage extinct than bacteria with SM resistance [13], this could have important implications for bacteria–phage coexistence.

To explore the long-term effects of phage challenge, we carried out evolution experiments where we repeatedly challenged populations of *P. aeruginosa* PA14 with phage DMS3vir, while monitoring the bacterial and phage population dynamics, and the composition and diversity of CRISPR arrays in the bacterial population along with the frequencies of an alternative resistance via the modification of the surface receptor to which the phage binds. We varied both the initial starting doses similarly to previous experiments [15], to assess the effect of associated infection-dependent costs, and frequency of reinfection to measure the populations’ robustness to infection over longer term ecologically relevant timeframes. We also investigated the genetics behind these types of resistance, combining whole-genome sequencing with deep sequencing of CRISPR arrays to provide a high-resolution description of the phenotypic and genotypic dynamics following phage infection.

## Results

### Initial dosage determines long-term dynamic

To study the dynamics of the acquisition and loss of CRISPR immunity in bacterial populations, we infected the model organism *P. aeruginosa* PA14 with phage DMS3vir [20]. DMS3vir is a mu-like phage [21] that uses the Type IV pilus of *P. aeruginosa* as its receptor. This phage carries a mutation in its repressor gene that prevents lysogeny, as well as a partial protospacer match, which promotes primed spacer acquisition by *P. aeruginosa* PA14 [19].

**Figure 1:**
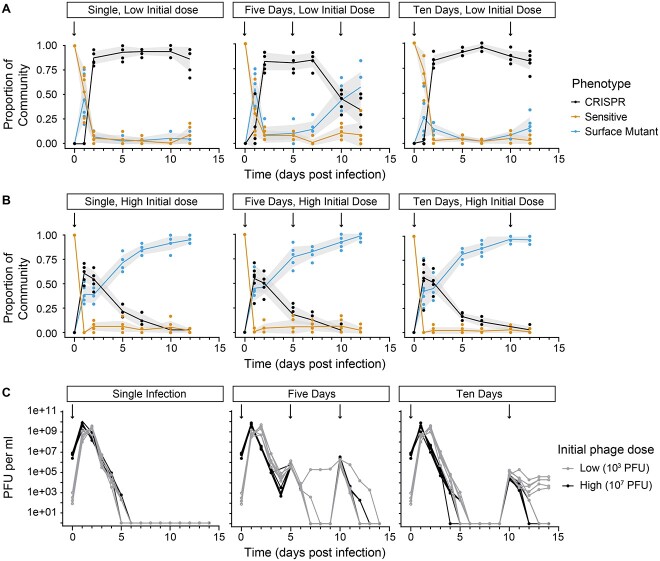
Population dynamics of phages and bacteria following infection with different phage doses; (A, B) fractions of sensitive (yellow), CRISPR immune (black), and surface mutant (SM) (blue) clones during the 15-day evolution experiment with (A) a low initial dose (10^3^ plaque-forming units (PFU)) or (B) a high initial dose (10^7^ PFU); black arrows indicate a (re)infection event (10^7^ PFU for both treatments, excluding the initial low-dose infections in (A); lines denote mean values of six independent biological replicate experiments and shaded areas represent ±1 SD; each colored circle reflects a sampling point where frequencies of sensitive, CRISPR immune, and SM were determined; (C) phage titers over time upon infection of *P. aeruginosa* PA14 with either 10^3^ PFU (low dose, gray line) or 10^7^ PFU (high dose, black line) of phage DMS3vir; each line represents an individual replicate population (*n* = 6); phage titers were recorded daily, and the limit of detection was 200 PFU/ml.

We aimed to assess how frequently CRISPR-based immunity is acquired over surface modification when phage exposure is varied and then track the subsequent population dynamics, using phenotypic characterization. A single initial exposure to a low phage dosage (10^3^ plaque-forming units [PFU]) led to high levels of CRISPR immunity (~90% of the population: 0.93 ± 0.06 [mean ± standard deviation], 5 days post-infection [DPI]) and low levels of surface mutants (0.03 ± 0.03, 5 DPI) and sensitive bacteria (0.04 ± 0.05, 5 DPI). This mixed population was relatively stable until the end of the experiment (12 days, Fig. 1A). In contrast, a higher initial phage dosage (10^7^ PFU) initially led to a mixed population of CRISPR-immune and surface mutant bacteria (~60:40 ratio [0.55 ± 0.09:0.39 ± 0.09, 2 DPI], respectively), followed by constant replacement of CRISPR-immune bacteria with surface mutants that were maintained long after the phage became extinct (Fig. 1B). The phage dynamics were similar in that both had peak population sizes of ~10^9^–10^10^ PFU/ml (low dose: 2.50 × 10^9^ ± 1.26 × 10^9^ PFU/ml, high dose: 6.00 × 10^9^ ± 3.62 × 10^9^ PFU/ml) followed by extinction; however, the low phage treatment lagged that of the high phage treatment, reaching its peak 2 DPI rather than 1 DPI (Fig. 1C). By 6 DPI in both treatments, the phage was extinct. This demonstrates that the initial level of phage infection, and subsequent timing of epidemic peak, determines the long-term trajectory of the population’s immune profile, even when the size of the phage epidemic is similar.

We next tested the robustness of the acquired CRISPR immunity across both dosages by reinfecting replicate populations with high phage doses at 5 and 10 DPI (Fig. 1A and B). The population dynamics of both reinfection treatments strongly mirrored those of the single infection at the high dose, with surface mutants increasing in frequency throughout the experiment (Fig. 1B). By contrast, in the low-dose, 5-day reinfection treatment, reinfection led to a subsequent decline in CRISPR-immune bacteria accompanied by replacement with surface mutant bacteria. In the 10-day reinfection treatment, we only observed a slight reduction in CRISPR-immune bacteria following reinfection, although the dynamics were trending in a similar direction to the 5-day reinfection treatment (Fig. 1A). Analysis of the final phenotypic frequencies at Day 12 found significant differences between phenotypes in all groups apart from the low-dosage, 5-day reinfection regime, which had an even distribution of each phenotype (Table S1). Phages were generally driven to extinction (Fig. 1C), with one exception: reinfection at Day 10, in the low-dosage regime, led to stable phage persistence at ~10^4^ PFU/ml. Taken together, these results strongly suggest that the initial window of exposure determines which resistance mechanism will prevail, in turn determining the robustness of the population to subsequent reinfection from the same phage, and that resistance is maintained long after the phage has become locally extinct.

### Sequencing reveals extensive spacer loss after infection

**Figure 2:**
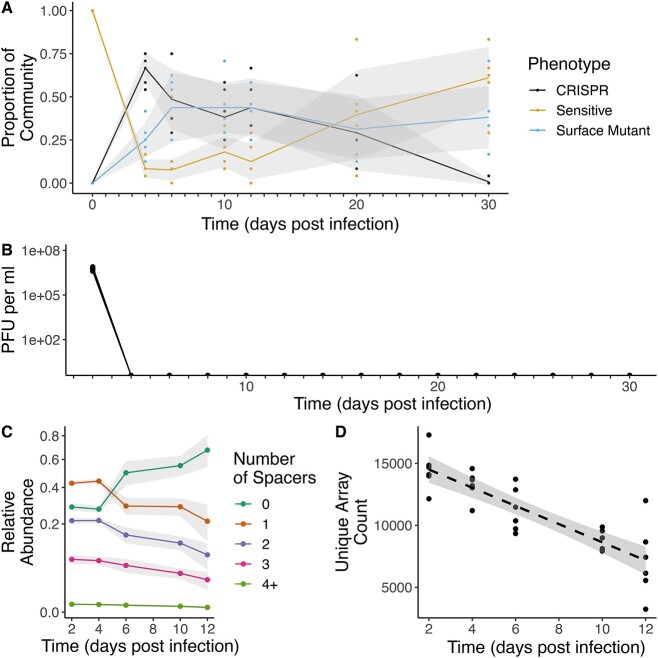
Population dynamics of bacteria and phage following infection; (A) frequencies of sensitive (yellow), CRISPR immune (black), surface mutants (SM) (blue), and SM clones during a 30-day evolution experiment following infection of *P. aeruginosa* PA14 with 10^7^ plaque-forming units (PFU) of phage DMS3vir; lines denote mean values of 6 independent biological replicate experiments and shaded areas represent ±1 SD; each colored circle reflects a sampling point where frequencies of sensitive, CRISPR immune, and SM were determined; (B) phage titers over time following infection of *P. aeruginosa* PA14 with 10^7^ PFU of phage DMS3vir; each black circle reflects a sampling point where phage titers were determined and the limit of detection is 200 PFU/ml; data show individual populations (*n* = 6); (C) frequencies over time of CRISPR arrays with no additional spacers compared to the ancestral WT PA14 genotype (dark green), one newly acquired spacer (orange), two acquired spacers (purple), three acquired spacers (pink), and four or more acquired spacers (light green); lines denote mean values of six independent biological replicate experiments and shaded areas represent ±1 SD; data are derived from amplicon sequencing of CRISPR arrays; (D) number of unique CRISPR arrays throughout the experiment identified via deep sequencing of array amplicons; unique arrays include unique spacers or unique combinations of spacers; shaded areas represent 95% confidence intervals and dashed line denotes linear model fit; data points represent individual populations (*n* = 6).

The dynamics we observed in the high phage dosage treatment revealed a transient period during which CRISPR-immune bacteria were more abundant than bacteria with surface mutations. In order to assess the genetic factors underlying this transition, we conducted a similar high-dosage experiment with an extended duration of 30 days. We then deep-sequenced the CRISPR arrays within the bacterial populations as well as the genomes of a surface mutant colony from each population. Bacteria and phage population dynamics were broadly similar to those that we observed before in the high phage dosage treatment (Fig. 2A and B).

Deep sequencing of CRISPR array amplicons (CRISPR1 and CRISPR2) from the evolved populations throughout the experiment revealed that populations rapidly acquired extremely high spacer diversity (16 270 ± 1762 [mean ± standard deviation] unique CRISPR arrays at 2 DPI). This spacer diversity was sufficient to cover all 5377 possible target sequences that are flanked by a conserved protospacer adjacent motif on the phage genome. Following this initial expansion of spacer content, arrays with one or more newly acquired spacers declined in frequency over time, whereas those that did not acquire new spacers during this experiment increased in frequency over time (GLM, *F*_1,89_ = 228, *P* < .0001; Fig. 2C), and this was associated with a steady decline in the population-level diversity of CRISPR arrays in terms of the absolute diversity (i.e. the number of unique CRISPR arrays in the population) (Fig. 2D). Simpson evenness increased over time, suggesting more equal frequencies of CRISPR arrays at the end of the experiment (Fig. S1), although by this point the abundance of CRISPR-immune bacteria was low. When we tracked the fates of individual spacers (in order to identify variation in spacer effectiveness), we typically found a consistent decline in their abundance, with only a few exceptions that most likely represent CRISPR-immune clones that subsequently acquired a beneficial mutation (Fig. S2).

### Reinfections reveal that CRISPR immunity is transient

**Figure 3:**
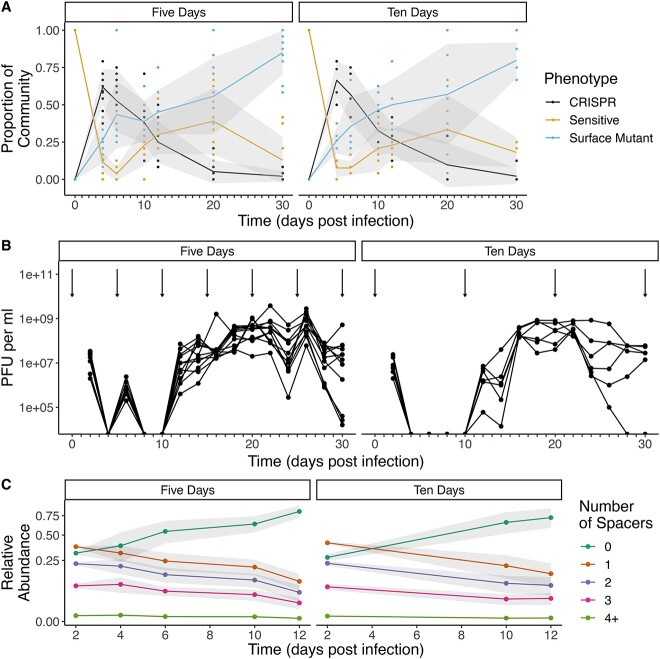
Population dynamics of bacteria and phage following reinfection; (A) frequencies of sensitive (yellow), CRISPR immune (black), and surface mutant (SM) (blue) clones during a 30-day evolution experiment following (re)infection of *P. aeruginosa* PA14 with 10^7^ plaque-forming units (PFU) of phage DMS3vir; lines denote mean values of 6 (5-day reinfection treatment) or 12 (10-day reinfection treatment) independent biological replicate experiments and shaded areas represent ±1 SD; each colored circle reflects a sampling point where frequencies of sensitive, CRISPR immune, and SM were determined; (B) phage titers over time upon (re)infection of *P. aeruginosa* PA14 with 10^7^ pfu of phage DMS3vir; arrows denote (re)infection events and each line represents an individual replicate population (*n* = 12 for 5-day reinfection treatment and *n* = 6 for 10-day reinfection treatment); phage titers were recorded every 2 days and the limit of detection is 200 pfu/ml; (C) frequencies over time of CRISPR arrays with no additional spacers compared to the ancestral WT PA14 genotype (dark green), one newly acquired spacer (orange), two acquired spacers (purple), three acquired spacers (pink), and four or more acquired spacers (light green); lines denote mean values of six independent biological replicate experiments and shaded areas represent ±1 SD; data are derived from amplicon sequencing of CRISPR arrays.

Similar to the experiment described above, when we reinfected these populations at 5 DPI, the phage was driven extinct rapidly in all six replicate populations, demonstrating that a sufficient proportion of the bacterial population remained phage resistant and that the levels of spacer diversity within the population remained sufficiently high to prevent bacteria–phage coexistence (Fig. 3A and B). When the same bacterial populations were reinfected 5 days later with the same phage (at 10 DPI), they had lost the ability to drive the phage extinct (Fig. 3B), and phage was able to persist for the remainder of the 30-day experiment in all replicates of the same treatment. This suggests that between 5 and 10 DPI, the proportion of CRISPR-immune bacteria or the diversity of spacers in the population became too low to drive the phage extinct. Consistent with this notion, the frequency of CRISPR-immune bacteria across both 5- and 10-DPI treatments was negatively correlated with phage titers (Pearson correlation coefficient = −0.68, *P* < .0001, *df* = 103, Fig. S3). In addition, reinfection at 10 DPI showed more variation between these experiments, suggesting that additional stochastic processes may determine phage persistence.

Deep sequencing of CRISPR arrays of these populations at 10 DPI identified an average of 10 779 (±2557, mean ± SD, *n* = 6) unique array sequences for the 5-day reinfection treatment and 8956 unique arrays (±2734, mean ± SD, *n* = 6) for the 10-day reinfection treatment. Similarly, CRISPR arrays with new spacers declined throughout the experiment (Fig. 3C). We have previously demonstrated that the presence of as few as 24 spacers is capable of consistently driving this phage to extinction [13]. Therefore, the levels of spacer diversity are vastly in excess of the levels required to drive phages extinct. However, these original results were obtained when CRISPR-immune clones made up 100% of the populations, whereas by Day 10 in this experiment, only ~25% of the population were CRISPR-immune bacteria. Therefore, the loss of population-level immunity is most likely driven by the decline in the proportion of CRISPR-immune bacteria in the bacterial population between the first reinfection (5 DPI) and the second reinfection (10 DPI).

To identify the genetic basis of the SM phenotype, a single SM clone was isolated from each population at 12 DPI and subjected to whole-genome sequencing. In all cases, SM resistance was associated with either a SNP in a pilus gene (*pilC* or *pilY1*) or the complete deletion of one (*pilM*) or more pilus genes, including a 10-kb deletion (Table S2). Competition experiments between the ancestral WT clone and evolved clones isolated at 4 DPI did not reveal fitness differences between the different phenotypes in the absence of phage (Fig. S4). Repeating these competitions with clones from Day 12 showed a general increase in fitness over time, although there were no differences between phenotypes within each sampling point (Table S3). This result suggests that surface modifications are associated with the modification or loss of the pilus, but that generally the spontaneous phage-resistance mutations that arise under the experimental conditions used here do not carry a detectable fitness cost in our assays. The observed long-term dynamics are therefore explained primarily by the initial benefit of each form of immunity, rather than any associated costs. Simply put, when phages are abundant, SM is more beneficial, and when phages are fewer, CRISPR is favored.

## Discussion

CRISPR-Cas systems have been identified in ~40% of bacterial and ~90% of archaeal genomes and can be grouped into two classes containing six types [22]. Despite the prevalence of these systems, the factors that determine the maintenance or loss of such acquired immunity are poorly understood. Experimental evolution studies of CRISPR-Cas are predominantly based on experiments with bacteria that carry Type I, Type II, or Type III systems (reviewed in Watson *et al.* [23]). Here, we study how the long-term dynamics of bacteria with type I CRISPR immunity depends on the initial phage dose, and its implications for bacteria–phage coexistence. We infected a susceptible population of PA14 with a lytic phage, DMS3vir, at either high or low phage dosage (10^3^ vs. 10^7^ PFU), triggering spacer acquisition and subsequent immunity. In both treatments, the phage was driven to extinction; however, the phenotypic composition of the populations varied markedly between treatments, with CRISPR immunity being maintained for longer in the low-dose treatment. The stability of CRISPR immunity in the low phage treatment suggests that spacers are not being lost at high frequencies from arrays, despite the observation that recombination between repeats can cause spacer loss (reviewed in Garrett [11]). By contrast, in the high-dose treatment, SM bacteria rapidly replace CRISPR bacteria. The speed at which SM invades is consistent with selection being the predominant driver of these dynamics. This is consistent with earlier studies that demonstrated that although high phage densities fuel spacer acquisition [15], SM will invade under high phage exposure due to costs associated with CRISPR immunity [16, 19]; therefore, the ratios of SM versus CRISPR immunity that emerge are dependent on phage exposure. In this experiment, although both treatments led to high phage titers, the timing differed, with the low-dose phage epidemic peaking a day later than the high phage dose. The lag in phage epidemic is consistent with the phenotypic data, as at 1 DPI, under low phage exposure, the population consisted of high levels of both sensitive and SM bacteria. By Day 2, these phenotypes had declined, presumably as sensitive cells were either lysed or acquired a spacer(s). Altogether, these results suggest that the combination of initial population size [15], phage density, and timing of epidemic peak will shape the long-term maintenance of CRISPR resistance. An important point to note is that although synergies between defenses can lead to more efficient resistance (reviewed in Tesson and Bernheim [24]), e.g. through the restriction-modification mediated generation of DNA substrate for CRISPR spacer acquisition [25], in this system, no synergy is observed. This is due to negative epistasis between spacer acquisition and surface modification, i.e. there is negligible benefit to carrying both types of resistance [13, 15].

Crucially, the invasion of SM following high initial phage exposure enabled stable coexistence between bacteria and phage because sensitive bacteria acted as a reservoir for phage amplification and the absence of CRISPR-immune bacteria prevented population-level immunity. Bacteria–phage coexistence was unlikely to be driven by mutations in the phage population. This is, first, because we reinfected with the ancestral phage and, second, because previous work has demonstrated that a diverse CRISPR population can overcome phage mutations [13] and our deep sequencing revealed extensive CRISPR diversity. Specifically, whereas Type I-F CRISPR-Cas immunity can be readily overcome through the acquisition of a point mutation in the corresponding target sequence on the phage genome if immunity is conferred by a single spacer, rapid acquisition of many different spacers by different bacteria in the population prevents the emergence and spread of such phage mutants [13, 26, 27]. By contrast, CRISPR–phage co-evolution where bacteria accumulate spacer(s) and phage accumulate escape mutations has been observed between *Streptococcus thermophilus*, which carries a Type II CRISPR-Cas system, and its phage 2972. Ultimately, the phage is driven extinct because spacer acquisition is virtually cost-free for the host [28, 29], whereas the phage faces an increasing cost of mutation accumulation that is compounded by diversification of CRISPR arrays in the bacterial population [26]. Within treatment (high vs. low phage exposure), dynamics were largely the same, despite phages persisting in some cases but not others (Figs 1A–C and 2A–C). This is likely due to resistance being cost-free in these experimental conditions; therefore, there is no selection against resistance once the phage has been removed from the community. This contrasts with previous experiments where we did observe a cost. There are likely many routes to resistance via mutation, or loss, of the pilus, all of which may shape the magnitude of associated costs. Under shaking conditions, attachment to a surface via the pilus is likely unnecessary, removing any such cost. In addition, the competition assays here were conducted using clones isolated from Days 4 and 12; therefore, any clone isolated must be sufficiently fit to be maintained in the experiment. In natural environments, the loss of the pilus is likely to carry higher costs. A previous study found that increased bacterial biodiversity amplified the costs associated with pilus loss [30] and future studies are required to directly assess such costs in natural environments.

By deep sequencing the CRISPR arrays and the genomes of SM bacteria, we could track the genetic basis of resistance. We observed that spacer diversity decreased in all treatments (2 and 3), which may explain the frequently observed conservation of the trailer-end of CRISPR loci [31], although other ecological and evolutionary processes, such as selective sweeps of multi-phage resistant CRISPR clones [32], are also likely important in natural communities. In *P. aeruginosa*, sequence analysis of CRISPR arrays from a cohort of Danish patients showed no within-patient spacer acquisition during up to 10 years of longitudinal sampling. Similarly, a collection of Russian clinical samples found identical arrays carried by different isolates [33] and a cohort of Greek isolates found identical CRISPR arrays within the same clonal complex [34]. In addition, global analysis of >700 *P. aeruginosa* isolate genomes found that local CRISPR diversity encompasses global diversity, with identical arrays found across continents and sampling times shared identical CRISPR arrays. This analysis also found that a subset of closely related temperate viruses are highly targeted by *P. aeruginosa* CRISPR arrays [35]. Taken together, these results suggest that spacer acquisition may be rare and limited to encounters with specific phage groups. Our results represent a scenario where one such phage is encountered and a priming spacer exists, which strongly increases spacer acquisition across diverse CRISPR systems [36, 37]. Moreover, our results likely reflect different temporal and spatial scales of diversity than the above studies. Additional studies focusing on the role of priming on the timescales of spacer acquisition and loss, as well as understanding why many bacteria possess multiple CRISPR arrays will be important for understanding the dynamics of spacer loss. For example, it has been speculated that the rate of spacer loss differs across arrays and therefore may represent “short-term memory” and “long-term memory” of previously encountered phages [9]. Lastly, understanding if this transient protection is also a phenomenon in systems that acquire spacers in positions other than the leader end of the array, such as *S. thermophilus* [38], will require future study.

The loss of spacer diversity over time could be relevant to phage therapy, which is receiving much interest due to the rise in antimicrobial resistance [39]. Long-lasting resistance mechanisms may severely hamper therapeutic usage of phages [40]. However, this work shows that CRISPR immunity may be transient, and that it may even be possible to take advantage of the loss of population-level CRISPR immunity to design well-timed reinfection schemes. For example, our study indicates that the dosage and timing of phage infections determine the level of resistance acquired. If the same processes occur in the clinic, then understanding which form of resistance is most likely to arise will be crucial for therapeutic success and require understanding of the interplay between bacterial host genetics and the phage used. The practical relevance of the multiplicity of infection used for phage therapy will also require further research, as our results suggest that high doses can select for different forms of immunity. Clinical trials have assessed cocktails of *Escherichia coli* and *P. aeruginosa* phages for treatment of burn wound patients [41] and experiments with murine models have demonstrated that phage therapy can provide highly effective treatment of *P. aeruginosa* infections [42-45]. A large clinical trial involving phages to treat *P. aeruginosa* infections in cystic fibrosis patients is also currently underway (ClinicalTrials.gov Identifier: NCT05453578). CRISPR systems are also being used to enhance phage therapy, either through the phage-mediated delivery of CRISPR systems that target the bacterial chromosome or through modification of phages with CRISPR engineering to increase efficacy (reviewed in Strathdee *et al*. [46]). An example of such enhancement is a phage that has been engineered to carry a mini-CRISPR array that targets the *Clostridium difficile* toxin locus [47]. Predicting the lifespan of these alternative technologies will require a thorough understanding of the ecology and evolution of the target host and enhanced phage. More generally, it remains to be seen whether the transient nature of CRISPR that we describe here will have a substantive impact in the clinic and biotechnology.

## Materials and Methods

### Bacterial strains and phages

The previously described *P. aeruginosa* strains UCBPP-PA14 and the isogenic mutant *csy3::LacZ* and phage DMS3vir have been previously described [20] and were used throughout this study.

### Evolution experiments

Evolution experiments were performed in six replicates by inoculating 6 ml M9 supplemented with 0.2% glucose with ~10^6^ colony-forming units of bacteria from fresh overnight cultures of the WT strain and adding 10^7^ or 10^3^ PFU of DMS3vir, followed by incubation at 37°C while shaking at 180 rpm. Cultures were transferred daily 1:100 for up to 30 days. Reinfections with ancestral phage (10^7^ PFU) were performed as follows: (a) bacterial cultures in the single infection treatment were only infected at 0 DPI; (b) bacterial cultures in the 5-day reinfection treatment were infected at 0, 5, 10, 15, 20, and 25 DPI; (c) bacterial cultures in the 10-day reinfection treatment were infected at 0, 10, and 20 DPI.

### Phage extraction and titration

Every second transfer, samples were taken just before transferring of the cultures into fresh medium. Phage was isolated from these samples using chloroform extractions. Next, phage was titrated by spotting serial dilutions of phage in M9 salts on a lawn of *P. aeruginosa* strain UCBPP-PA14 *csy3::LacZ* bacteria for quantification.

### Immunity and resistance profiling

Bacterial immunity against the ancestral phage was determined by streaking individual clones (24 clones per sample) through ancestral phage DMS3vir and phage DMS3vir carrying the anti-CRISPR F1 (*acrIF1*) gene as described previously [13]. Bacterial clones sensitive to both phages were scored as “sensitive,” those resistant to DMS3vir but sensitive to DMS3vir + AcrIF1 were scored as “CRISPR immune,” and bacterial clones resistant to both phages were scored as “surface mutants.” CRISPR-Cas-mediated immunity was further confirmed by PCR using primers CTAAGCCTTGTACGAAGTCTC and CGCCGAAGGCCAGCGCGCCGGTG (for CRISPR 1) and GCCGTCCAGAAGTCACCACCCG and TCAGCAAGTTACGAGACCTCG (for CRISPR 2). Surface modification was further confirmed on the basis of colony morphology (phage DMS3vir is pilus specific, therefore surface mutants have motility defects, resulting in a modified colony morphology) and a lack of new CRISPR spacers. From these analyses, fractions of each phenotype (sensitive, CRISPR immune, surface mutant) were calculated for each replicate experiment. The resistance phenotypic assays were conducted with colonies sampled directly from experimental cultures or recovered from cryopreserved samples (Fig. 1A, B 1 DPI, 2A 30 DPI, and Fig. 3A 30 DPI).

### Deep sequencing analysis

Full bacterial genomic DNA was isolated using the Qiagen QIAmp DNA mini kit as per the manufacturer’s protocols. A PCR amplification was performed for both CRISPR arrays (CRISPR1 and CRISPR2, see Table S4 for sequences and PCR conditions). PCR reactions contained 5 μl DreamTaq master mix (ThermoScientific, UK), 0.5 μl forward primer, 0.5 μl reverse primer, 1.5 μl MilliQ water, 0.5 μL DMSO, and 2 μl template DNA. Sample purity was determined by NanoDrop and DNA concentrations were quantified using a Qubit fluorometer (ThermoFisher, UK).

### CRISPR array sequencing protocol

Two separate CRISPR primer (CRISPR1 and CRISPR2 locus) pairs were designed for two first rounds of PCR. Two microliters of template DNA entered a first round of PCR. The primer design incorporates a recognition sequence to allow a secondary nested PCR process. Samples were first purified with Ampure SPRI Beads before entering the second PCR performed to incorporate Illumina adapter sequences. Samples were purified using Ampure SPRI Beads before being quantified using Qubit and assessed for size distribution using the Fragment Analyzer (Agilent). Successfully generated amplicon libraries were taken forward and pooled in equimolar amounts, then size selected with a Pippin Prep machine (Sage Science) using a range of 180–600 bps. The quantity and quality of each pool was assessed by Bioanalyzer and subsequently by qPCR using the Illumina Library Quantification Kit from Kapa on a Roche Light Cycler LC480II according to the manufacturer’s instructions. Template DNA was denatured according to the protocol described in the Illumina cBot User guide (Illumina Document #15006165 v05) and loaded at 12.5 pM concentration. Fragmented PhiX phage genome was added to the sequence library at 15% in order to increase the sequence complexity. The sequencing of each pool was carried out on one lane of an Illumina MiSeq, at 2×250 bp paired-end sequencing with v2 chemistry.

### Bioinformatics analysis

#### Sequence quality control

Base-calling and de-multiplexing of indexed reads were performed by CASAVA version 1.8.2 (Illumina) to produce 97 samples from each of the 2 lanes of sequence data. FASTQ files were trimmed to remove Illumina adapter sequences using Cutadapt version 1.2.1 [48]. The option “-O 3” was set, so the 3′ end of any reads that matched the adapter sequence over at least 3 bp was trimmed off. The reads were further trimmed to remove low-quality bases, using Sickle version 1.200 [49] with a minimum window quality score of 20. After trimming, reads shorter than 10 bp were removed. The raw reads were subjected to a Cutadapt trimming step to remove PCR primer sequences that could potentially introduce an artificial level of complexity in the samples. To improve base quality in both read pairs, sequencing errors were corrected in both forward and reverse reads using the error-correct module within SPAdes sequence assembler, version 3.1.0 [50]. Read pairs were aligned to produce a single sequence for each pair that would entirely span the amplicon using PEAR (version 0.9.10; [51]). In addition, sequences with uncalled bases (Ns) were removed. To remove sequences originating from potential PCR primer dimers or from any spurious amplification events, a size selection was applied to each merged sequence set, respectively between 30 and 140 bp for CRISPR1 and 70–500 bp for CRISPR2. To remove any “bleed through” of PhiX sequences, each sample was compared with the complete PhiX sequence (GenBank gi9626372) using BLASTN [52]. Sequences matching PhiX (E-value <10^−5^) were filtered out of the dataset.

#### Clustering and diversity metrics

For each dataset, any sequences passing the filters (from any sample) were merged into a single file. This final sequence file, plus its metadata file describing each sample, was used for the analysis by using a custom pipeline based on QIIME 1.9.0 [53]. Clusters were defined using SWARM [54], using the strictest (default) parameters. This tool aggregates a sequence to a cluster if the sequence shows similarity with any of the sequences already present in that cluster. The similarity threshold is not fixed but defined within the dataset. A minimum cluster size filter is applied to retain clusters containing at least two sequences and potential chimeric sequences due to PCR events were discarded as well. To calculate the abundance of each cluster, sequences were then aligned on the centroid sequence identified for each clusters, using a minimum similarity threshold of 99% for the entire length of the sequence using the “usearch_global” function in VSEARCH.

The sequencing depth of all samples was explored using the “Chao1” [55] richness index plotted as a rarefaction curve. Counts in the cluster abundance tables were repeatedly subsampled (rarefied; 33 repetitions) at sampling depths of 1000, 12 000, 22 000, … 150 000. The average Chao1 value obtained by repeating the test 33 times is assigned as alpha-diversity at that specific number of reads for that sample implemented in QIIME. Because all samples reached a clear asymptote, i.e. no samples were under-sampled with regard to spacer diversity, rarefaction was not applied. An abundance table for each locus was used to estimate the richness and evenness of the samples using the following estimators: total observed sequence variants, Shannon index, Simpson diversity, and Simpson evenness. Each of these metrics was determined using QIIME function alpha_diversity.py.

Spacers were extracted from the CRISPR arrays via CRISPRdetect [56] and mapped to the phage genome using bwa [57] and samtools [58].

#### Whole-genome sequencing

All populations were plated at 12 DPI and 12 colonies were screened for their phage-resistance phenotype, as described previously [13]. Of these, a single clone with surface modification (SM)-based resistance was selected. Each colony was then suspended in 100 μl H_2_O and streaked across a lysogeny broth (LB) agar plate. Cells were scraped from these plates and added to bead-beating tubes prior to shipping to the MicrobesNG service (Birmingham, UK). As an additional control, the ancestral PA14 laboratory stock was included. Genomic DNA libraries were prepared using Nextera XT Library Prep Kit (Illumina, San Diego, USA) and sequenced on the Illumina HiSeq using a 250-bp paired-end protocol by MicrobesNG (Birmingham, UK). Reads were adapter trimmed using Trimmomatic 0.30 with a sliding window quality cutoff of Q15 [59]. The ancestral control was initially mapped to the PA14 reference genome (NC_008463) using the Breseq pipeline (version 0.32.0, [60]). The reference genome was then modified to match the identified mutations (to account for divergence between the laboratory strain and original reference genome) using the “gdtools” function. The remaining samples were then mapped against this modified reference and alignments were used for identifying mutations.

### Competition assays

For each population, a single clone of each phenotype (a CRISPR-immune, a SM-resistant, and a sensitive clone) was picked (where possible) and competed against a LacZ marked reference strain. Populations were serially transferred with a 1:100 dilution in M9 media supplemented with 0.2% glucose. Populations were plated at *T* = 0 and after 3 days on LB agar supplemented with 30 μg of X-gal to determine the relative frequencies of the evolved clone and the reference strain. Relative fitness values were determined as described in van Houte *et al.* [13]: relative fitness = [(fraction strain A at *t* = *x*) × (1 − (fraction strain A at *t* = 0))]/[(fraction strain A at *t* = 0) × (1 − (fraction strain A at *t* = *x*))].

### Statistical analyses

General linear models were used using the appropriate error structure, and model residuals were assessed for model fit. Significance was determined following stepwise deletion of terms or through Tukey post hoc testing with adjustment for multiple comparisons. General linear mixed-effect models were used for the competition assays, with population specified as a random effect, due to the nonindependence of clones isolated from the same populations. Mixed-effect modeling was carried out using the “lme4” package with post hoc testing conducted with the “emmeans” package. Correlations between the frequency of each phenotype and the PFU per milliliter during the evolution experiment were tested with a Pearson correlation using the 5- and 10-day reinfection regimes using all sampling points between Days 4 and 30 (4, 6, 10, 12, 20, and 30 DPI). Analysis of phenotype frequencies in the phage dosage experiment used binomial regression with data grouped by dose and reinfection regime. All statistical analysis was conducted in R (version 4.0.5).

## Author contributions

Edze R. Westra, Stineke van Houte, Sean Meaden, Angus Buckling, and Bridget N.J. Watson designed experiments. Loris Capria, Sean Meaden Bridget N.J. Watson and Benoit J. Pons performed the experiments under supervision of Stineke van Houte and Edze R. Westra. Ellinor O. Alseth prepared samples for sequencing. Sean Meaden, Luca Lenzi, and Angus Buckling performed sequencing data analyses. Edze R. Westra and Sean Meaden wrote the manuscript.

## Conflicts of interest

E.R.W. is inventor on patent GB2303034.9.

## Funding

This work was funded by a grant from the European Research Council under the European Union’s Horizon 2020 research and innovation programme (ERC-STG-2016-714 478 to E.R.W.). E.R.W. further acknowledges the Natural Environment Research Council (NE/M018350/1) and the Biotechnology and Biological Sciences Research Council (BB/N017412/1) for funding. B.N.J.W. acknowledges support from the Biotechnology and Biological Sciences Research Council (BB/X010600/1). S.M. acknowledges support from the BBSRC (BB/X009793/1). A.B. acknowledges the Natural Environment Research Council, the Biotechnology and Biological Sciences Research Council and the Royal Society, the Leverhulme Trust, and the AXA research fund for funding. S.V.H. acknowledges funding from BBSRC (grant number BB/R010781/1). Whole-genome sequencing was provided by MicrobesNG (http://www.microbesng.uk), which is supported by the BBSRC (grant number BB/L024209/1). RNA-sequencing was conducted at the University of Exeter sequencing center, which is supported by the Wellcome Trust.

## Data availability

Sequence data are available from the European Nucleotide Archive under study number PRJEB44752.

## Supplementary Material

Supplementary_Information_revision1_wrad039
