# Supplementary material for: CRISPR-Cas in Pseudomonas aeruginosa provides transient population-level immunity against high phage exposures
Source: ISME J. 2024 Jan 10;18(1):wrad039. doi: 10.1093/ismejo/wrad039 (PMC10873826; doi:10.1093/ismejo/wrad039)
Supplement: Supplementary_Information_revision1_wrad039 [file supplementary_information_revision1_wrad039.docx]

**Supplementary Information**

**Fig S1. CRISPR array diversity declines through the experiment.**

Diversity metrics of CRISPR array genetic diversity for the 5 and 10 day reinfection regimes. Metrics are derived from deep sequencing of array amplicons and in all cases array diversity reached a clear asymptote therefore no subsampling was performed. Dashed lines represent linear model fits and shaded areas represent 95% confidence intervals.

**Fig S2. CRISPR arrays with newly acquired spacers decline in frequency.**

Frequencies (cut-off at 0.001%) of individual CRISPR arrays throughout the experiment. Green line denotes the ancestral array genotype; black lines represent arrays with 1 or more acquired spacers. Panel numbers denote the experimental replicate. Fig. 1D shows the total frequencies of arrays carrying 0-4 spacers, whereas these plots show the frequencies of individual arrays.

**

**Fig. S3. Correlations between phenotypes and phage titres.**

Phenotype proportions correlated against the phage titre present at the same sampling time. Dashed lines represent a linear model fit and shaded areas represent 95% confidence intervals. Each point represents a population and sampling time point. Data are taken from five and ten-day reinfection regimes using all sampling points between day 4 and 30 (4, 6, 10, 12, 20 and 30 dpi). A) Surface mutant bacteria, B) CRISPR-immune bacteria, C) Sensitive bacteria.

**Fig S4. Relative fitness of clones in the absence of phage infection**

Fitness of CRISPR immune clones (grey), sensitive bacteria (yellow) and bacteria with SM resistance (blue) isolated at day 4 of the evolution experiment, relative to the ancestral WT PA14 strain. Each point represents a clone isolated from the single infection treatment (n = 6 per phenotype).

**Table S1. Model summaries of statistical analysis of end point phenotypes**

Summaries of binomial regression GLMs modelling the variation in phenotype frequencies (CRISPR, Sensitive, Surface Mutation) at the end of the dosage experiment (day 12). Data were grouped by phage infection dosage (high or low) and the reinfection frequency (single, every 5 days or every 10 days) and modelled individually.

| Phage Dose | Reinfection Frequency | Residual DF | Residual Deviance | p-value |
| --- | --- | --- | --- | --- |
| low | 1 | 17 | 13.58 | **<0.01** |
| low | 5 | 17 | 5.17 | 0.1845 |
| low | 10 | 17 | 11.62 | **<0.01** |
| high | 1 | 17 | 18.42 | **<0.0001** |
| high | 5 | 17 | 21.07 | **<0.001** |
| high | 10 | 17 | 19.69 | **<0.0001** |

**Table S2.** **Pilus mutations and deletions lead to a phage-resistant phenotype.**

Whole genome sequencing of surface mutants (1 colony per population) revealed that phage resistance is conferred by mutations in pilus-associated genes. Sample labels represent experimental treatments (D = Daily reinfection, S = Single infection, F = Five day reinfection, T = Ten day reinfection) and replicate populations (1-6/12). Mutations also present when mapping the ancestral PA14 sample back to the reference are excluded.

| sample | position | mutation | description |
| --- | --- | --- | --- |
| S1 | 5,236,592 | G→A | type 4 fimbrial biogenesis protein pilC |
| S2 | 5,948,050 | Δ10,288 bp | Pilus genes deletion |
| S3 | 5,948,050 | Δ10,288 bp | Pilus genes deletion |
| S4 | 5,948,050 | Δ10,288 bp | Pilus genes deletion |
| S5 | 5,948,050 | Δ10,288 bp | Pilus genes deletion |
| S6 | 5,948,050 | Δ10,288 bp | Pilus genes deletion |
| F1 | 5,948,050 | Δ10,288 bp | Pilus genes deletion |
| F2 | 5,948,050 | Δ10,288 bp | Pilus genes deletion |
| F3 | 5,948,050 | Δ10,288 bp | Pilus genes deletion |
| F4 | 5,948,050 | Δ10,288 bp | Pilus genes deletion |
| F5 | 5,948,050 | Δ10,288 bp | Pilus genes deletion |
| F6 | 5,948,050 | Δ10,288 bp | Pilus genes deletion |
| F7 | 5,948,050 | Δ10,288 bp | Pilus genes deletion |
| F8 | 5,373,594 | C→T | type 4 fimbrial biogenesis protein PilY1 |
| F9 | 5,948,050 | Δ10,288 bp | Pilus genes deletion |
| F10 | 5,948,050 | Δ10,288 bp | Pilus genes deletion |
| F11 | 5,373,594 | C→T | type 4 fimbrial biogenesis protein PilY1 |
| F12 | 5,948,050 | Δ10,288 bp | Pilus genes deletion |
| T1 | 5,948,050 | Δ10,288 bp | Pilus genes deletion |
| T2 | 5,950,888 | Δ3,261 bp | pilM deletion |
| T3 | 5,948,050 | Δ10,288 bp | Pilus genes deletion |
| T4 | 5,948,050 | Δ10,288 bp | Pilus genes deletion |
| T5 | 5,948,050 | Δ10,288 bp | Pilus genes deletion |
| T6 | 5,948,050 | Δ10,288 bp | Pilus genes deletion |

**Table S3**

Post-hoc comparisons of contrasts that compare relative fitness. Numbers in the contrast denote sampling time (day 4 or day 12) and the given phenotypes (CRISPR, SM or sensitive).

| Contrast | Estimate | SE | df | t.ratio | p-value |
| --- | --- | --- | --- | --- | --- |
| 4_CRISPR-12_CRISPR | -0.2773 | 0.0474 | 122.8124 | -5.8475 | **1e-04** |
| 4_CRISPR-4_Sens | -0.1115 | 0.0586 | 109.4472 | -1.9017 | 0.4067 |
| 4_CRISPR-12_Sens | -0.3887 | 0.0742 | 111.9035 | -5.2423 | **1e-04** |
| 4_CRISPR-4_SM | -0.1243 | 0.0516 | 96.3502 | -2.4093 | 0.1634 |
| 4_CRISPR-12_SM | -0.4015 | 0.0693 | 111.6448 | -5.7951 | **1e-04** |
| 12_CRISPR-4_Sens | 0.1658 | 0.0766 | 120.3732 | 2.1639 | 0.2625 |
| 12_CRISPR-12_Sens | -0.1115 | 0.0586 | 109.4472 | -1.9017 | 0.4067 |
| 12_CRISPR-4_SM | 0.153 | 0.0708 | 113.2484 | 2.1593 | 0.2652 |
| 12_CRISPR-12_SM | -0.1243 | 0.0516 | 96.3502 | -2.4093 | 0.1634 |
| 4_Sens-12_Sens | -0.2773 | 0.0474 | 122.8124 | -5.8475 | **1e-04** |
| 4_Sens-4_SM | -0.0128 | 0.058 | 108.8156 | -0.2211 | 0.9999 |
| 4_Sens-12_SM | -0.2901 | 0.0754 | 119.9723 | -3.8455 | **0.0026** |
| 12_Sens-4_SM | 0.2644 | 0.0744 | 112.3086 | 3.5541 | **0.0072** |
| 12_Sens-12_SM | -0.0128 | 0.058 | 108.8156 | -0.2211 | 0.9999 |
| 4_SM-12_SM | -0.2773 | 0.0474 | 122.8124 | -5.8475 | **1e-04** |

**Table S4.**

Primers and PCR conditions used for amplicon sequence analysis:

| CRISPR array | Primers | Sequence | PCR conditions |
| --- | --- | --- | --- |
| CRISPR 1 | CR1_F  CR1_R | GGCGCTGGAGCCCTTGGGGCTTGG  GCGGCTGCCGGTGGTAGCGGGTG | 95 C X 1 minutes  95 C X 15 seconds  69 C X 15 seconds  72 C X 30 seconds  72 C X 7 minutes |
| CRISPR 2 | CR2_F  CR2_R | GCTCGACTACTACAACGTCCGGC  GGGTTTCTGGCGGGAAAAACTCGG | 95 C X 1 minutes  95 C X 15 seconds  69 C X 15 seconds  72 C X 30 seconds  72 C X 7 minutes |
